# Multiple cavernous hemangiomas of the skull with dural tail sign: a case report and literature review

**DOI:** 10.1186/1471-2377-13-155

**Published:** 2013-10-25

**Authors:** Peng Xu, Shengyong Lan, Youming Liang, Quan Xiao

**Affiliations:** 1 Department of Neurosurgery, The People’s Hospital of Guangxi Zhuang Autonomous Region, 06 Taoyuan Road, 530021, Nanning, China

**Keywords:** Cavernous hemangiomas, Skull, Orbit

## Abstract

**Background:**

Primary intraosseous cavernous hemangioma is a rare bony tumor. To date, only 9 cases of multiple lesions and 2 cases with a dural tail sign have been reported.

**Case presentation:**

Here, we present a case of multiple cavernous hemangiomas of the skull with dural tail sign in a 24-year-old man. No abnormalities were observed in the right orbit by craniography, but frontal bone destruction was unintentionally discovered. Computed tomography and magnetic resonance imaging demonstrated multiple intraosseous lesions that destroyed the surrounding bone and intracranial extension. Total resection of the two lesions and cranioplasty were performed. Histological examination confirmed the lesions as a cavernous hemangioma.

**Conclusion:**

Cavernous hemangioma is a rare bony tumor that should be considered in the differential diagnosis of skull tumors. Resection of all lesions should be performed on patients with multiple cavernous hemangiomas, and these patients should have regular follow-up examinations. Based on this case, and our literature review, we found that outcomes are usually very good.

## Background

Primary intraosseous cavernous hemangioma (PICH) is a benign vascular lesion. More than 50 percent of PICHs are observed in the spine, whereas these lesions seldom occur in the skull. PICHs account for 0.2 percent of bone tumors and 7 percent of skull tumors [1]. Skull PICHs are usually isolated lesions protruding into the external plate of the skull, and they most commonly involve the parietal and frontal bone, but rarely involve the orbits [1]. We report a rare case of skull PICH. It presented multiple lesions and grew into the internal skull with a dural tail sign.

## Case presentation

**Figure 1:**
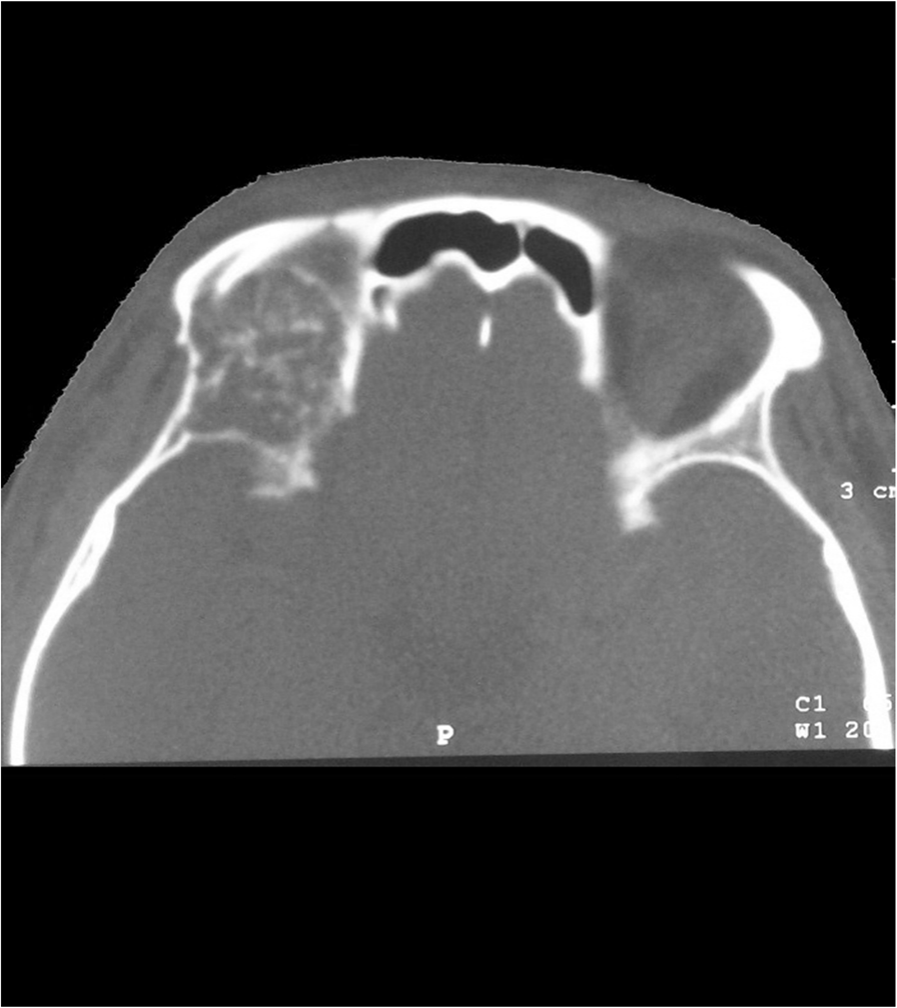
Bone window computed tomography scan demonstrating a 3 cm mass destroying the surrounding bone and penetrating into the internal skull in the upward wall of the right orbit.

**Figure 2:**
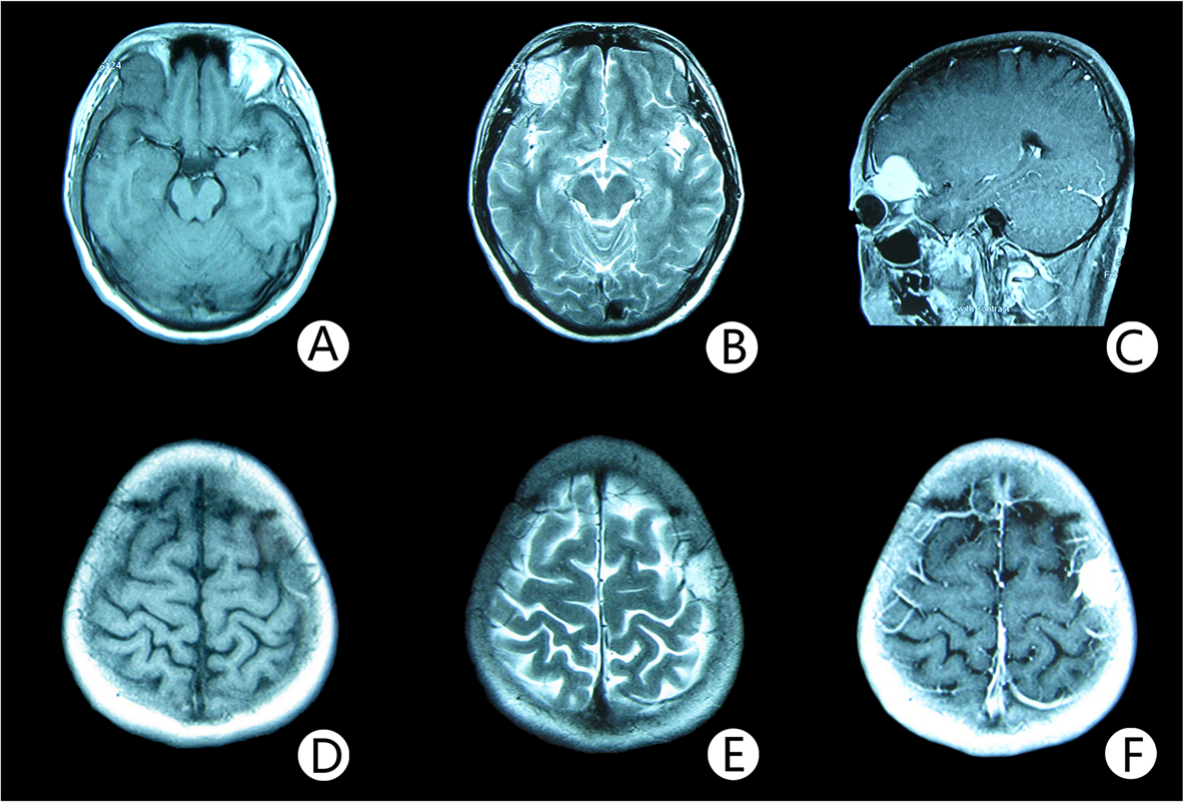
**Magnetic resonance image features of the two lesions.** Noncontrast axial T1-weighted MR image **(A)** reveals a well circumscribed mass, measuring 3 cm × 3 cm × 3 cm, in the upward wall of the right orbit with intracranial extension. The lesion is well defined, and the signal intensity is of mixed intensity with some central hyperintensity. T2-weighted MR image **(B)** shows a lesion of high signal intensity. There is no surrounding edema. After administration of gadopentetate dimeglumine **(C)**, strong and homogeneous enhancement of the mass and a dural tail sign is observed. The lesion extended intracranially and appears to compress the underlying brain. The MR image of the mass in the frontal bone over the central sulcus **(D; E; F)** is similar to the one in the upward wall of the right orbit **(A; B; C)**.

A 24-year-old man presented with progressive right eye exophthalmous for one year, without any other ocular or nervous symptoms. There were no obvious abnormalities in the right orbit; however, left frontal bone destruction was found on plain radiographs of the skull. To obtain more information, orbital computed tomography (CT) scanning was conducted, and a lesion was observed in the right orbital roof (Figure 1). The lesion was approximately 3 cm × 3 cm × 3 cm with regular edges. It was destroying the surrounding bone and penetrating into the internal skull. Magnetic resonance imaging (MRI) showed that the two lesions in the upward wall of the right orbit and left frontotemporal bone were similar. They both penetrated into the internal skull, whereas the external skull was not involved. MR imaging revealed T1-isointense and T2-hyperinstense circumscribed lesions. These uniformly enhancing lesions were associated with an enhancing dural tail (Figure 2). A surgical plan was made under the assumption that these lesions were meningiomas.

**Figure 3:**
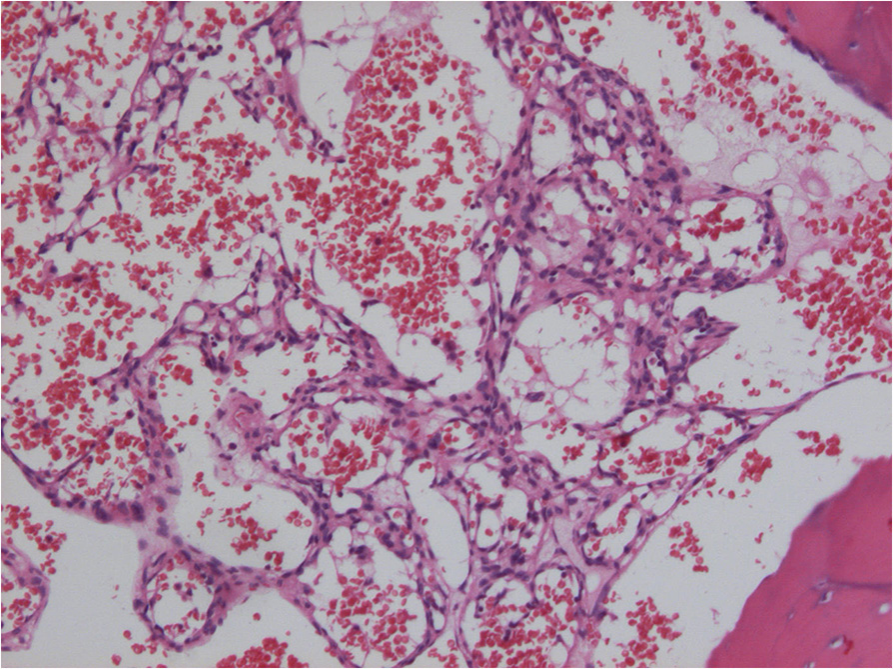
**Photomicrograph of the resected specimen showing erythrocytes filling many sinusoidal channels lined with a single layer of endothelial cells.** Hematoxylin and eosin staining, ×100.

During the operation, a grid-like boney tissue was observed within these red-colored tumors, which adhered themselves to bloody meninges. The two lesions were completely removed along with the normal bone tissue around them. After the operation, the diagnosis of skull cavernous hemangioma was made by histopathological review (Figure 3). The immunohistochemical staining for CD34, CD31, P53, and Ki-67 were all positive.

## Discussion and conclusions

In 1845, Toynbee first reported a case of cavernous hemangioma of the skull, and this condition was defined by Rowbotham from the histological point of view until 1924 [2]. Most reviews of PICH of the skull concern the parietal and frontal bone. Only 45 cases referred to the orbital bone, but they were all single lesions described in a review by Madge et al. [2]. Therefore, cases of multiple lesions are rare. So far, only 10 cases of multiple lesions have been reported (including this case) [3]. Skull PICHs are characterized by protrusion into the external plate of the skull, and the cases in which these tumors grow into the internal skull are rare. To date, only three cases have been reported (including this case) [4,5].

Radiography of the skull is the most useful method to identify PICHs, whose typical features are a “honeycomb” or “sunray” pattern [1]. However, in our case, the lesions in the parietal bone on plain film were atypical, showing osteolysis and cortical bone expansion. This appearance has been reported in cases of radiographically occult orbital lesions. Owing to the complexity of the orbit, plain radiographs are occasionally misleading, and orbital CT is recommended. With computed tomograms, one can visualize the honeycomb pattern of boney trabeculae and design an operation for complete resection [1]. MR imaging can clearly show the relationship between the tumor and brain tissue. T1- and T2-weighted MR have different performances. T1-weighted MR is mainly influenced by fat content, so it is nonhomogeneously isointense and generally resembles the gray matter, whereas the T2-weighted MR is hyperintense and may be associated with water and slow-flowing blood in the tumor. In our case, due to the small size of the lesions in the parietal bone, these characteristics were not evident. We did observe T1- and T2- hypointense signals at the center of the orbital lesions, and both lesions enhanced, consistent with prior reports [4,6].

There are two histological classifications of PICH: sinus and capillary. Most PICHs of the spinal canal are classified as sinus, and many PICHs of the skull are classified as capillary [7]. Madge et al. summarized 45 cases of orbital PICHs, of which 80 percent were sinus, 17 percent were capillary, and 3 percent were mixed types [2]. Sinus and capillary PICHs have different characteristics. Capillary hemangiomas lack fibrous septa and have smaller vascular lumens. The endothelial cells are often small and lack mitotic activity. In contrast, sinus hemangiomas tend to relapse, and are composed of groups of large, dilated blood vessels are separated by fibrous tissue. Surgery for cavernous hemangiomas is likely to be complicated by hemorrhage [8].

The present case should be distinguished from multiple tumors that penetrate into the internal skull. Because approximately 70 percent of meningiomas have the dural tail sign and because the other MRI features of this case were similar to those of meningiomas, we considered this to be the disease before the operation [9]. In this case, the CT bone window was atypical for meningioma, however. The other differential diagnoses included hemangiopericytoma, osteosarcoma, metastases, and inflammatory granuloma [6].

Total surgical resection is the major treatment of skull PICH. This surgical procedure has the benefits of low blood loss during the operation and a low recurrence rate after the operation [10]. Resection should include a 1-cm width of the normal skull around the lesions. For frequently occurring skull PICHs, it is controversial whether the resection should be conducted in conjunction with the resection of the asymptomatic lesions or not. Among the nine cases of multiple PICHs previously reported, six cases were treated with total resection, and in one case the asymptomatic lesions were removed. Naama et al. [10] hypothesized that asymptomatic lesions were observed because PICH grows slowly, and its symptoms often appear several years later. However, cranial tumors can cause neurological symptoms and harbor a small risk of hemorrhage [6]. We planned on an operative resection of meningiomas, but concluded that gross total resection would have been our goal had we known the diagnosis *a priori*.

Other reported treatments include curettage and radiotherapy. Curettage may lead to serious bleeding during the operation and recurrence after the operation [11]. Radiotherapy is only able to prevent the tumor from growing, and cannot eradicate the lesions. In addition, malignant transformation of PICHs after radiotherapy has been reported [6,11]. Therefore, these two methods should only be applied in cases in which it is not completely safe to perform removal. Although PICH is rare, it should be considered in the identification of lesions occupying the skull space. For frequently occurring cases with the dural tail sign, surgery should strive to remove all of the lesions. After several years of development, asymptomatic lesions may grow into the internal skull, leading to symptoms of the nervous system.

## Consent

Written informed consent was obtained from the patient and his parents for publication of this case report and any accompanying images. A copy of the written consent is available for review by the series editor of this journal.

## Abbreviations

PICH: Primary intraosseous cavernous hemangioma; MRI: Magnetic resonance imaging; CT: Computed tomography.

## Competing interests

We confirm that we have read the Journal’s position on issues involved in ethical publication and affirm that this report is consistent with those guidelines. None of the authors have any competing interests to disclose.

## Authors’ contributions

All authors fulfill the authorship criteria because of their substantial contributions to the conception, design, analysis, and interpretation of the data. XP analyzed the data and drafted the manuscript. LSY and LYM participated in data acquisition. XQ conceived the study, and participated in its design and in data acquisition. All authors read and approved the final manuscript.

## Pre-publication history

The pre-publication history for this paper can be accessed here:

http://www.biomedcentral.com/1471-2377/13/155/prepub
